# The Cellular and Biophysical Origins of Microvascular Vasomotion: A Scoping Review

**DOI:** 10.7759/cureus.107580

**Published:** 2026-04-23

**Authors:** Alexis C Spencer-Vargas, Coleman Nelson, London Danchulis, Yama Xu, Luca Walborn, Harvey N Mayrovitz

**Affiliations:** 1 College of Medicine, Nova Southeastern University Dr. Kiran C. Patel College of Osteopathic Medicine, Davie, USA; 2 College of Medicine, Osteopathic Medical School, Nova Southeastern University Dr. Kiran C. Patel College of Osteopathic Medicine, Davie, USA; 3 Foundational Sciences, Nova Southeastern University, Clearwater, USA; 4 College of Medicine, Osteopathic Medical School, Nova Southeastern University Dr. Kiran C. Patel College of Osteopathic Medicine, Clearwater, USA; 5 Medical Education, Nova Southeastern University Dr. Kiran C. Patel College of Allopathic Medicine, Davie, USA

**Keywords:** arteriolar smooth muscle, blood flow regulation, calcium oscillations, endothelial modulation, hemodynamic oscillations, microcirculation, microvascular physiology, microvascular vasomotion, neurovascular coupling, vascular tone regulation

## Abstract

Microvascular vasomotion involves spontaneous rhythmic changes in arteriolar diameter that contribute to local perfusion and oxygen delivery. Although this phenomenon has long been recognized, the mechanisms underlying the initiation and coordination of these oscillations remain incompletely defined. A focused synthesis of recent mechanistic studies helps to better elucidate how vasomotion is initiated and regulated across vascular beds. Our objective was to review and integrate current evidence on the cellular, molecular, and biophysical mechanisms underlying microvascular vasomotion, with a focus on intrinsic smooth muscle oscillatory behavior and the factors that influence its expression. A scoping review was conducted using Web of Science, PubMed, Embase, and Ovid MEDLINE. English language studies published from 2005 onward were considered, and earlier foundational reports were included when directly relevant to mechanistic interpretation. Experimental, clinical, and modeling studies addressing the origins or regulation of vasomotion in microvessels were eligible. From 958 retrieved records, duplicates were removed, and titles and abstracts were screened. Full-text assessment identified 30 studies for inclusion in the data synthesis. Considering a range of experimental models and vascular regions, the results suggested a common mechanistic theme. Oscillatory intracellular calcium activity within arteriolar smooth muscle cells was most consistently associated with the generation of vasomotor rhythms and periodic shifts in vascular tone. Electrophysiologic studies indicated that membrane potential behavior, ion channel function, and intercellular coupling were important in determining the timing and coordination of these oscillations. Signals arising from endothelial and astrocytic pathways were repeatedly shown to modulate oscillation amplitude and regional responsiveness, whereas neural and sympathetic inputs altered oscillatory strength in response to changing physiological demands. Mechanical influences, including shear forces and network geometry, contributed to the organization and propagation of oscillations through the microvascular network. Under hypoxic or ischemic stress, vasomotor activity often intensified, and altered patterns were described in conditions such as peripheral arterial disease and cerebrovascular dysfunction. It is concluded that the existing evidence supports a model in which microvascular vasomotion originates primarily from intrinsic oscillatory behavior of arteriolar smooth muscle cells, with endothelial, neural, metabolic, and mechanical factors shaping amplitude, synchronization, and spatial expression. The variability in study design, measurement approaches, and frequency band definitions remains a challenge to cross-study comparison. Future investigations that incorporate standardized analytical methods and in vivo approaches to link cellular oscillations to network-level flow dynamics may help clarify the physiological and clinical significance of vasomotion.

## Introduction and background

One definition of the microvasculature refers to it as a network of blood vessels approximately 20 µm or less in diameter, composed of arterioles, capillaries, post-capillary venules, and their associated cellular components [1]. Others suggest that diameter alone is insufficient to specify, but if done, then such a definition might include vessels about 100 µm or less [2]. The microvascular network tightly regulates oxygen delivery and the exchange of solutes, fluids, and hormones [1]. Within this network, arterioles have been noted to exhibit apparently spontaneous oscillations in their diameter, a process referred to as vasomotion [3-6]. A consequence of this vasomotion is a waxing and waning of blood flow in accordance with the vasomotion phases that has been termed "flowmotion" [7,8]. These blood flow variations are often observed when skin is measured with laser Doppler flowmetry [9]. 

The phenomenon of vasomotion is not a new one, having been documented in the 1850s by observation of rhythmic dilation and contraction of veins that occur independently of heart contractility within bat wing circulation [10]. Despite having been observed over a century ago, the precise mechanisms and physiological implications of vasomotion remain incompletely understood. Initially, various hypotheses regarding the origin of vasomotion [11-13] and its physiological effects [14-17] were proposed. Some current evidence suggests that these oscillations may optimize tissue perfusion, protect against ischemia, and regulate local blood flow distribution [18]. At the cellular level, vasomotion is thought to arise from intracellular Ca²⁺ oscillations, with gap junctional coupling enabling coordination across the vascular network. These processes contribute to rhythmic contraction and relaxation of arteriolar smooth muscle cells, resulting in periodic changes in vessel diameter and vascular tone [19]. 

Recent studies have emphasized the relevance of altered vasomotion in cerebral pathology, including ischemic stroke [19], Alzheimer’s disease [20], and stress-related vascular changes [21], as well as systemic conditions such as diabetes [20] and hypertension [22], underscoring its clinical relevance. Although progress has been made, a synthesis of the past two decades of research on the origins and physiological effects of vasomotion is lacking. The aim of this review is to synthesize current knowledge on the cellular and biophysical origins of microvascular vasomotion, highlighting both established mechanisms and areas that remain unresolved. Thus, the specific objective of this scoping review is to synthesize current evidence on the cellular, molecular, and biophysical origins of microvascular vasomotion. We aim to identify studies that experimentally or computationally discuss the mechanisms by which vasomotion arises primarily in arteriolar segments of the microvasculature, including ion channel dynamics, endothelial-smooth muscle coupling, gap junctional communication, and intrinsic oscillatory behavior. Studies limited to descriptive, functional, or pathological effects of vasomotion will be excluded. 

For the purposes of this review, the microvasculature is defined as vessels ≤100 µm in diameter, encompassing terminal arterioles, capillaries, and post-capillary venules. This broader definition was used to ensure inclusion of resistance arterioles, which contain vascular smooth muscle cells and are generally considered a principal site of vasomotion generation [3,11]. Narrower definitions limited to smaller vessels may exclude these functionally important segments and overlook key mechanistic studies relevant to the origin of vasomotion. Accordingly, when referring to the microvascular network in this review, mechanistic interpretations of vasomotion are attributed primarily to arteriolar segments unless otherwise specified.

## Review

Methods 

Study Design and Protocol 

This scoping review was conducted using the Joanna Briggs Institute (JBI) methodology for scoping reviews and adheres to the Preferred Reporting Items for Systematic Reviews and Meta-Analyses extension for Scoping Reviews (PRISMA-ScR) guidelines [23]. 

Research Question 

The review was guided by the following question: *What cellular, molecular, and biophysical mechanisms give rise to spontaneous rhythmic diameter oscillations (vasomotion) in microvascular vessels (≤100 µm)?*

Eligibility Criteria 

This review included experimental (in vivo, in vitro, ex vivo), clinical, and computational studies that examined the mechanisms underlying microvascular vasomotion. Studies published in English from 2005 onward were considered to capture contemporary evidence generated using modern imaging and molecular techniques. Earlier studies were included when they provided foundational mechanistic insights. Studies focused solely on descriptive or clinical manifestations of vasomotion without addressing underlying mechanisms were excluded. 

Search Strategy 

A comprehensive literature search was conducted across four electronic databases: Web of Science, PubMed, Embase, and Ovid MEDLINE. Search strategies were designed to identify studies examining the cellular, molecular, and biophysical mechanisms of microvascular vasomotion. Detailed search strings are provided in Table 1. 

**Table 1:** Boolean Search Strategies

Database	Search Strategy	Limitations
PubMed	(“microcirculation”[MeSH] OR arteriole* OR capillar* OR microvascular OR “post-capillary venule*”) AND (“vasomotion” OR “vascular oscillation*” OR “rhythmic diameter” OR “microvascular oscillation” OR flomotion OR flowmotion) AND (“origin” OR mechanism OR physiology OR perfusion OR “blood flow regulation” OR “oxygen delivery”)	English language; 2005–present; reference lists of included studies were hand-searched.
Ovid MEDLINE	(exp Microcirculation/ OR arteriole*.mp. OR capillar*.mp. OR microvascular.mp. OR post-capillary venule*.mp.) AND (vasomotion.mp. OR vascular oscillation*.mp. OR rhythmic diameter.mp. OR microvascular oscillation.mp. OR flomotion.mp. OR flowmotion.mp.) AND (origin.mp. OR mechanism.mp. OR physiology.mp. OR source*.mp. OR affect*.mp. OR generat*.mp.)	Limited to English; publication years 2005–current.
EMBASE	(‘microcirculation’/exp OR microcirculation OR arteriole* OR capillar* OR microvascular OR “post-capillary venule*”) AND (‘vasomotion’/exp OR vasomotion OR “vascular oscillation*” OR “rhythmic diameter” OR “microvascular oscillation” OR flomotion OR ‘flowmotion’/exp OR flowmotion) AND (‘origin’/exp OR origin OR ‘mechanism’/exp OR mechanism OR ‘physiology’/exp OR physiology OR ‘perfusion’/exp OR perfusion OR ‘blood flow regulation’/exp OR “blood flow regulation” OR ‘oxygen delivery’/exp OR “oxygen delivery”)	English language; 2005–2025.
Web of Science	TS=(microcirculation OR microvascular OR arteriole* OR capillar* OR "post-capillary venule*") AND TS=(vasomotion OR "vascular oscillation*" OR "rhythmic diameter" OR "microvascular oscillation" OR flomotion OR flowmotion) AND TS=(origin OR mechanism* OR physiology OR perfusion OR ischemia OR "blood flow regulation" OR "oxygen delivery")	English; document type: article; years 2005–present.

Selection of Source of Evidence

All identified records were imported into EndNote (Version 21), and duplicates were removed. Titles and abstracts were independently screened by reviewers against predefined inclusion criteria. Full-text articles were then assessed for eligibility by two independent reviewers, with discrepancies resolved through discussion or by a third reviewer. 

Data Charting Process 

Data were extracted independently by two reviewers using a standardized data charting form. Extracted information included study design, experimental model or population, vascular bed, mechanistic focus, and key findings. Data management and screening were conducted using Rayyan [24] and Microsoft Excel. 

Critical Appraisal of Sources of Evidence 

Methodological quality was assessed using JBI Critical Appraisal Tools appropriate to each study design. Three reviewers independently evaluated the included studies, and discrepancies were resolved through consensus. Studies were categorized as low (>70%), moderate (50-70%), or high (<50%) risk of bias. In keeping with scoping review methodology, no studies were excluded based on quality, though findings from higher-risk studies, as indicated in Table 2, were interpreted with caution.

**Table 2:** Summary of included studies examining the cellular and physiological mechanisms underlying microvascular vasomotion. Risk-of-bias assessment: Low risk (>70% of criteria met), moderate risk (50–70%), and high risk (<50%)

Author Year	Study Aim	Model & Vascular Bed	Design or Methods	Main Findings	Limitations	Ris-of-Bias Assessment
Smooth Muscle Ca 2+ Oscillations and Ionic Mechanisms						
Colantuoni et al., 1984 [3]	Quantitation of rhythmic diameter changes	Hamster arterioles	Intravital microscopy	Vasomotion persisted for 10–14 days in intact microcirculation. Oscillations were localized to individual arteriolar segments (not globally synchronized). Oscillation frequency increased as vessel diameter decreased. Strong inverse correlation between vessel diameter and frequency (r = 0.894).	Small n	Moderate
Li et al., 2024 [25]	To determine whether intracellular Ca²⁺ oscillations in arteriolar smooth muscle cells drive spontaneous vasomotion and how ischemia affects this mechanism	In vivo mouse model; cerebral arterioles.	Simultaneous measurement of arteriolar diameter and smooth muscle Ca²⁺ oscillations at baseline and after transient ischemia.	Demonstrated that smooth muscle Ca²⁺ oscillations are necessary for reperfusion after ischemia. Loss of rhythmic Ca²⁺ activity leads to "no-reflow," and pharmacologic restoration of Ca²⁺ oscillations reestablishes flow.	Animal model; cerebral circulation only; short-term ischemia model.	Low
Borysova et al., 2013 [26]	Investigate how Ca²⁺ signaling in smooth muscle cells and pericytes coordinates vasomotion within intact microvascular networks	Rat ureter microcirculation	Live confocal imaging of intracellular Ca²⁺ dynamics in smooth muscle cells and pericytes during vasoconstrictor stimulation	Coordinated Ca²⁺ oscillations were associated with rhythmic microvascular constrictions. Higher-frequency oscillations produced stronger and more synchronized diameter changes, whereas low-frequency oscillations were weak and poorly coordinated.	Ex vivo preparation; pharmacologic stimulation model; non-cerebral vascular bed.	Moderate
Broegger et al., 2011 [27]	Evaluate Bestrophin-3 role in vasomotion	In vivo rat mesenteric small arteries	siRNA-mediated downregulation of bestrophin-3 with assessment of vasomotion, contractility, and intracellular Ca²⁺ synchronization	Bestrophin-3, a Ca²⁺-activated chloride channel, is essential for rhythmic vasomotion. Silencing the channel abolishes oscillations and disrupts vessel rhythmicity.	siRNA specificity limits	Low
Yang et al., 2015 [28]	To investigate how Kir channel–dependent membrane properties influence smooth muscle electrical behavior in arterioles	Isolated guinea pig arterioles (spiral modiolar, cerebral, mesenteric).	Intracellular electrophysiologic recordings of resting membrane potential with pharmacologic Kir channel blockade	Kir2.x channel density creates bistable membrane potentials in vascular smooth muscle cells, enabling rapid transitions between constricted and dilated states. This bistability facilitates regenerative vasomotion and propagation along the arteriole.	Ex vivo	Low
Lapi et al., 2017 [29]	Characterize spontaneous rhythmic diameter oscillations in rat pial arterioles	In vivo rat pial arterioles (cerebral microcirculation)	Intravital fluorescence microscopy with spectral analysis under baseline conditions and pharmacologic endothelial modulation	Low-frequency oscillations were present across arteriolar orders. Endothelial inhibition reduced oscillation amplitude but did not abolish rhythmicity, indicating modulation rather than generation of vasomotion.	Animal model; pharmacologic manipulation; cerebral vascular bed only.	High
Endothelial and Astrocytic Control of Vasomotion						
Haidey et al., 2021 [30]	To determine whether astrocytes contribute to the generation or modulation of cerebral vasomotion	Acute brain slices and awake mice; cortical arterioles	Two-photon imaging with astrocyte-specific COX-1 manipulation and calcium signaling modulation	Astrocytes generate ultra-slow arteriole oscillations through TRPV4 activation and COX-1–mediated prostanoid release. Blocking astrocytic pathways suppresses vasomotion.	Off-target effects	Low
Rossi et al.,2005 [31]	To characterize cutaneous flowmotion patterns in peripheral arterial obstructive disease (PAOD)	Human lower-limb cutaneous microcirculation (PAOD patients and healthy controls)	Laser Doppler flowmetry with generalized wavelet spectral analysis at baseline and during post-occlusive reactive hyperemia	Distinct low-frequency oscillatory components were identified. PAOD patients exhibited increased oscillatory power in smooth muscle, endothelial, and sympathetic frequency bands at rest but showed impaired augmentation during reactive hyperemia.	Indirect spectral inference; cutaneous circulation only; observational clinical design.	Moderate
Tikhonova et al., 2022 [32]	To examine phase interactions between heart rate variability, respiration, and peripheral microhemodynamic oscillations	Human upper and lower extremity cutaneous microcirculation	Simultaneous recording of laser Doppler microvascular flow, heart rate variability, and respiratory signals with phase interaction analysis	Peripheral microvascular oscillations exhibited structured phase relationships with systemic physiological rhythms, suggesting organized but non-random coupling between central and peripheral oscillatory processes.	Indirect flow-based measurements; correlational design; no direct cellular or mechanistic assessment.	Moderate
Neural, Sympathetic, and Hemodynamic Drivers						
Vetri et al., 2007 [33]	To assess how cortical activation influences pial arteriolar vasomotion	In vivo rat cerebral pial arterioles	Sciatic nerve stimulation with intravital imaging of pial arteriolar diameter oscillations	Neurovascular activation altered oscillatory patterns in region-specific manner. Vasomotion persisted under basal conditions, suggesting neural input modulates but does not generate rhythmic activity.	Anesthesia suppresses rhythms	Low
Sakurai et al., 2006 [34]	To determine how sympathetic nerve stimulation affects flowmotion and tissue fluid exchange	In vivo rabbit ear microcirculation	Laser Doppler flowmetry during electrical cervical sympathetic nerve stimulation and temperature manipulation	Sympathetic stimulation induces vasomotion and dramatically enhances tissue–capillary fluid exchange, demonstrating a functional purpose for oscillatory behavior.	Anesthesia-heavy	Low
Wu et al., 2020 [35]	To evaluate microvascular oscillatory responses of the plantar skin during walking at different intensities	Human plantar skin microcirculation	Laser Doppler flowmetry with wavelet spectral analysis during treadmill walking at graded speeds	Walking-induced neural and metabolic activation modulates multiple vasomotion bands, especially myogenic and neurogenic oscillations.	Small sample	Moderate
Vasomotion Coordination, Synchronization, and Network Dynamics						
Zhang et al., 2024 [36]	To characterize cerebral vasomotion dynamics and their modulation following stroke	In vivo mouse cerebral pial arterioles	High-resolution diameter measurements with simultaneous assessment of cerebral blood flow and autonomic indices	Using high-speed Ca²⁺ imaging, demonstrated precise temporal coupling between smooth muscle Ca²⁺ spikes and arteriolar diameter changes, and showed network desynchronization after stroke.	Complex methodology	Low
Trzeciakowski et al., 2008 [37]	To evaluate the nonlinear and chaotic properties of coronary flowmotion	In vivo canine coronary microcirculation	Fluorescent flow tracking with spectral analysis under nitric oxide synthase inhibition and purinergic blockade	Flowmotion exhibited nonlinear, chaotic characteristics. Disruption of nitric oxide signaling altered spectral distribution but did not eliminate oscillatory behavior.	Animal model; coronary circulation only; indirect flow-based assessment.	Low
Tigno et al., 2011 [38]	To assess changes in vasomotion complexity during progression of diabetes	Non-human primate cutaneous microcirculation	Laser Doppler flowmetry with nonlinear dynamic analysis.	Found a progressive loss of randomness (more deterministic vasomotion) in diabetic monkeys, indicating pathology-induced alterations in network dynamics.	Disease model; cutaneous circulation only.	Moderate
Liu et al., 2022 [39]	To investigate whether low-frequency vasomotion (~0.01–0.2 Hz) arises from heartbeat period variability and vascular nonlinear dynamics	In vivo human radial artery and simulated small arterial network models measured via laser Doppler flowmetry	Wavelet and Hilbert–Huang spectral analyses using single-tube and multi-connected tube models.	Low-frequency oscillations (0.01–0.2 Hz) persisted in reconstructed signals derived from heartbeat variability, and nonlinear vascular network models generated additional low-frequency components, particularly at branch points; the authors propose that vasomotion may emerge from the interaction between heartbeat variability and vascular nonlinearity.	Did not directly measure smooth muscle Ca²⁺ activity, and relied on simplified computational geometries.	Moderate
Hald et al., 2019 [40]	To determine how stimulation timing influences vasomotor responses	Ex vivo rat mesenteric arterioles	Sequential vasoconstrictor and vasodilator stimulation with force and oscillation assessment.	Stimulation history significantly altered vasomotion amplitude and force responses, indicating time-dependent modulation of oscillatory behavior.	Ex vivo preparation;	Low
Vasomotion During Hypoxia, Ischemia, Stroke, and Metabolic Stress						
Salvi et al., 2018 [41]	To assess the effect of hypoxia and ischemia on slow-wave vasomotion	Human cutaneous microcirculation (lowlanders and high-altitude natives)	Laser Doppler flowmetry with spectral analysis at varying altitudes and during arterial occlusion	High-altitude hypoxia significantly increased slow-wave vasomotion amplitude. Ischemia further amplified oscillatory power, suggesting hypoxia enhances rhythmic microvascular activity.	Small, heterogeneous cohorts	Moderate
Goltsov et al., 2017 [42]	To characterize oscillatory blood flow changes in acute ischemic stroke.	Human cerebral microcirculation (acute ischemic stroke patients)	Laser Doppler flowmetry with spectral and computational modeling analysis	Stroke altered oscillatory patterns, with increased myogenic frequency activity and hemispheric asymmetry in vasomotion.	Clinical observational study; indirect spectral inference; small cohort.	Moderate
Tikhomiro et al., 2009 [43]	To evaluate microcirculatory oscillations and blood rheology in cerebrovascular disorders	Human cutaneous microcirculation (stroke patients and controls)	Laser Doppler flowmetry and rheological assessment.	Stroke patients exhibited reduced perfusion but increased oscillatory variability and amplitude in endothelial, neurogenic, and myogenic bands.	Indirect oscillation measurement; observational design.	Moderate
Thorn et al., 2011 [44]	To investigate the relationship between vasomotion and oxygen extraction	Human skin microcirculation	Combined optical reflectance spectroscopy and laser Doppler flowmetry	Low-frequency vasomotion was temporally associated with fluctuations in oxygen saturation, supporting a link between oscillatory flow and oxygen extraction.	Small sample	Low
Marcinek et al., 2025 [45]	To determine whether baseline myogenic oscillations predict hypoxic microvascular responsiveness	Human cutaneous microcirculation (healthy and cardiovascular disease populations)	Flow-mediated skin fluorescence with spectral analysis before and after induced hypoxia	Baseline myogenic oscillatory strength predicted hypoxic response capacity. Reduced baseline oscillations were associated with impaired adaptation.	Cutaneous vascular bed; indirect spectral inference; observational design.	Moderate
Mechanical, Shear, and Structural Drivers of Vasomotion						
Widmer et al., 2006 [46]	To examine shear-mediated mechanisms underlying heat-induced microvascular flow responses	In vivo Pallid bat wing microcirculation	Intravital microscopy during local heating with neural blockade and nitric oxide synthase inhibition.	Identified a shear-mediated biphasic response in bat skin microcirculation following heat exposure, driven by changes in endothelial shear forces.	Animal model; thermoregulatory context; non-mammalian species.	Low
Widmer et al., 2008 [47]	To identify the spatial origin of heat-induced oscillatory flow responses	In vivo Pallid bat wing microcirculation	Intravital diameter measurements of upstream and downstream microvessels during heating trials	Upstream microvessels exhibited phase-leading oscillations relative to downstream branches, suggesting hierarchical coordination within the network.	Animal model; thermoregulatory context; non-mammalian species.	Low
Xu et al., 2013 [48]	To assess the effects of static magnetic field exposure on impaired vasomotion	Rat caudal artery ligation model	Near-infrared spectroscopy with Fourier analysis of vasomotion under ligation and magnetic field exposure	Vasomotion amplitude peaked at low frequencies and was elevated after ligation; magnetic field exposure reduced oscillation amplitude without altering mean perfusion.	Mechanism unclear	Low
Conceptual, Computational, and Theoretical Models						
Koenigsberger et al., 2006 [22]	To determine how arterial wall stress influences the emergence and characteristics of vasomotion	Computational model of small arterial smooth muscle	Biophysical modeling incorporating transmural pressure, wall stress, and intracellular Ca²⁺ dynamics.	Oscillatory behavior emerged within specific pressure and agonist ranges. Wall stress influenced oscillation frequency and stability, suggesting mechanical forces contribute to vasomotion generation.	No empirical validation	High
Gonzalez et al., 1994 [12]	Model of arterial vasomotion	To model the origin and dynamics of vasomotion in small arteries	Ion-based modeling of membrane potential dynamics incorporating calcium and potassium conductance	Interactions between voltage-dependent Ca²⁺ channels and potassium currents generated periodic membrane depolarizations, producing rhythmic contractile activity consistent with intrinsic smooth muscle oscillators.	No direct validation	High
Jacobsen et al., 2007 [49]	To model synchronization mechanisms among smooth muscle cells in the arterial wall	Computational model of arterial smooth muscle cell networks	Mathematical modeling of SMC coupling via gap junctions and intracellular Ca²⁺ dynamics.	cGMP signaling can synchronize SMC oscillators	No empirical data	High
Arciero et al., 2012 [50]	To determine whether mechanical feedback mechanisms can generate spontaneous microvascular oscillations	Mathematical model of single microvessel diameter regulation	Computational modeling incorporating wall tension, shear stress, and metabolic feedback.	Oscillations emerged from interactions between vessel wall mechanics and active tone regulation, even without predefined cellular oscillators.	Single-vessel computational model; simplified assumptions.	High
Pradhan et al., 2009 [51]	To evaluate how synchronization patterns influence tissue oxygenation	Computational skeletal muscle microvascular network model	Simulation of interacting vasomotor units with varying synchronization states.	Partial desynchronization enhanced simulated oxygen delivery compared with fully synchronized oscillations.	Theoretical only	High

Results

Study Selection and Characteristics 

A total of 958 records were identified through the initial search. After removal of 386 duplicates, 572 records were screened by title and abstract, of which 508 were excluded based on predefined eligibility criteria. Sixty-four full-text articles were assessed, with 34 excluded for not addressing the mechanistic origins of vasomotion or not meeting the inclusion criteria. A total of 30 studies were included in the final synthesis. 

**Figure 1:**
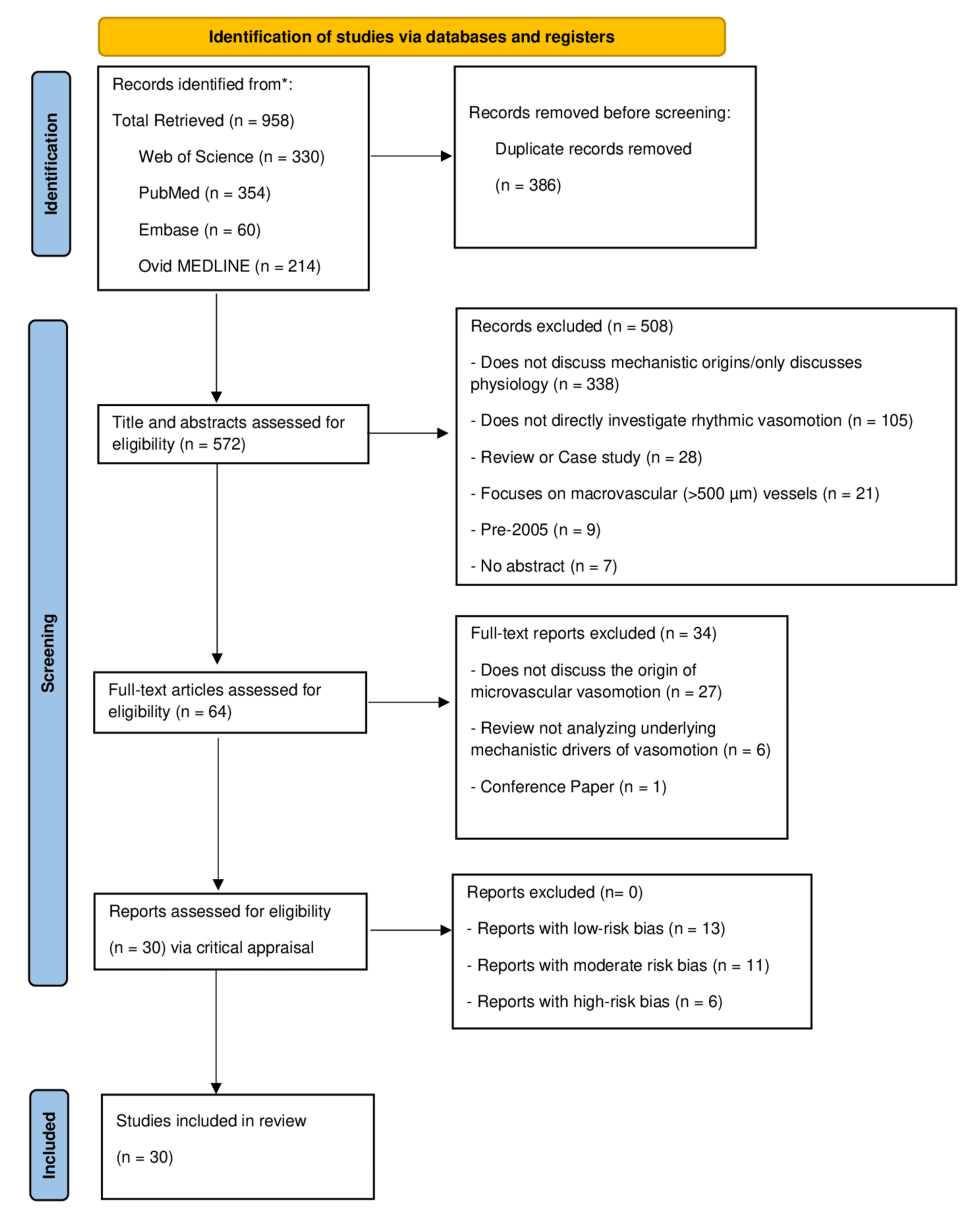
Preferred Reporting Items for Systematic Reviews and Meta-analyses extension for scoping review (PRISMA-ScR) flow diagram

The search was limited to studies published from 2005 onward to capture contemporary evidence, with earlier foundational studies included where relevant. The study selection process is summarized in the Preferred Reporting Items for Systematic Reviews and Meta-analyses extension for scoping review (PRISMA-ScR) flow diagram (Figure 1). 

Findings were synthesized narratively, with studies grouped according to shared mechanistic themes and methodological approaches. A quantitative meta-analysis was not performed due to substantial heterogeneity in study design, measurement techniques, and outcome variables. The included studies varied in vascular beds, experimental models, and analytical approaches. A summary of study characteristics and key findings is provided in Table 2.

Arteriolar Smooth Muscle Dynamics 

Early in vivo evidence characterizing the origin of microvascular vasomotion was provided by Colantuoni et al. [3], who used long-term intravital microscopy to examine spontaneous diameter oscillations in the hamster cheek pouch microcirculation. Vasomotion was observed continuously for up to two weeks in unanesthetized animals, supporting the idea that rhythmic microvascular activity is an intrinsic property of intact vessels rather than a response to external stimuli. Oscillations were localized to individual arteriolar segments rather than occurring uniformly across the network, suggesting a locally generated origin. 

Across arteriolar orders, smaller vessels exhibited higher oscillation frequencies, indicating an inverse relationship between vessel diameter and oscillatory activity. Changes in frequency at branch points and downstream propagation of oscillatory waves further support the presence of segmental control rather than centralized coordination. 

Pharmacological interventions targeting smooth muscle activity resulted in marked changes in vessel diameter and disruption of normal oscillatory patterns, reinforcing the role of arteriolar smooth muscle cells as key drivers of vasomotion. Collectively, these findings support the concept that vasomotion arises from locally generated oscillatory activity within arteriolar smooth muscle, with frequency and propagation characteristics influenced by vessel size and network structure. 

*Calcium Oscillations and Ionic Mechanisms*​​​

Subsequent studies using high-resolution imaging further clarified the cellular mechanisms underlying vasomotion. Using in vivo two-photon microscopy in mouse cerebral arterioles, Li et al. [25] demonstrated rhythmic diameter oscillations under baseline conditions that were present in arterioles but absent in venules, highlighting the role of arteriolar segments in generating vasomotion. Following transient cerebral ischemia, vasomotion frequency decreased, and oscillatory timing became more irregular, indicating disruption of normal rhythmic activity under pathophysiologic conditions. 

Using smooth muscle-specific calcium imaging, the same study showed that these diameter oscillations are driven by rhythmic intracellular Ca²⁺ activity within arteriolar smooth muscle cells. Ischemia was associated with reduced Ca²⁺ oscillation frequency, decreased amplitude, and increased variability, along with an overall reduction in cytosolic Ca²⁺ levels. Restoration of Ca²⁺ oscillatory activity coincided with recovery of vasomotion, while mean vessel diameter remained unchanged. These findings suggest that Ca²⁺ oscillations primarily regulate rhythmic diameter fluctuations rather than baseline vascular tone. 

Similar findings were reported by Borysova et al. [26], who used live confocal imaging of intact ureteric microvascular networks to demonstrate coordinated Ca²⁺ oscillations and rhythmic constrictions in response to vasoconstrictor stimulation (arginine, vasopressin, and phenylephrine). Lower-frequency Ca²⁺ signals were associated with weak and poorly coordinated contractions, whereas higher-frequency oscillations produced stronger and more synchronized vasomotor responses. Together, these studies support a frequency-dependent relationship between intracellular Ca²⁺ dynamics and the strength and coordination of vasomotion. 

Molecular Regulation of Vasomotion

Additional insight into the coordination of smooth muscle Ca²⁺ activity has been provided by molecular manipulation studies. Using in vivo siRNA-mediated downregulation of bestrophin-3 in rat mesenteric small arteries, Broegger et al. [27] demonstrated that vasomotion depends on intrinsic smooth muscle mechanisms that are distinct from those regulating tonic contraction. Reduction of bestrophin-3 expression did not alter baseline vessel diameter or contractile responsiveness, indicating that overall vascular tone remained preserved. 

Despite preserved tonicity, suppression of bestrophin-3 led to a marked reduction in vasomotion amplitude without affecting oscillation frequency. Calcium imaging revealed that this effect was associated with loss of synchronization of intracellular Ca²⁺ oscillations across smooth muscle cells, resulting in more disorganized and asynchronous activity. 

Pharmacologic restoration of signaling pathways partially recovered oscillatory behavior but did not fully restore vasomotion amplitude, suggesting that bestrophin-3 plays a key role in coordinating smooth muscle cell activity required for effective vasomotion. Together, these findings support the concept that vasomotion depends not only on the presence of Ca²⁺ oscillations, but also on their synchronization across the vascular wall. 

Membrane Electrophysiology

Electrophysiological studies have examined intrinsic SMC membrane properties associated with rhythmic behavior. Using intracellular recordings from isolated arteries and arterioles, Yang et al. [28] identified vessel-specific differences in resting membrane potential, with some arteriolar beds exhibiting relatively depolarized baseline states compared with others. 

Within individual SMC populations, resting membrane potentials displayed a bimodal distribution, suggesting the presence of distinct electrical states within the same vessel. This behavior was not explained by differences in ion channel expression, as levels of inwardly rectifying potassium (Kir) channels were similar across vessel types. 

However, pharmacological blockade of Kir channel activity altered membrane potential and disrupted this bimodal distribution, indicating that functional properties of membrane conductance, rather than channel abundance, play a key role in shaping intrinsic electrical behavior. These findings suggest that variability in SMC membrane potential may contribute to the initiation and modulation of rhythmic vasomotor activity. 

Microvascular Network Dynamics 

At the microvascular network level, Lapi et al. [29] used intravital fluorescence microscopy to characterize spontaneous vasomotion in rat pial arterioles. Rhythmic diameter oscillations were observed across all arteriolar orders under baseline conditions, indicating that vasomotion is a widespread feature of the microvascular network. Oscillation amplitude was more pronounced in resistance-sized arterioles compared with larger vessels, suggesting a greater functional role in regulating local blood flow. 

Spectral analysis revealed a dominant low-frequency component consistent with smooth muscle-driven activity across vessel types. Pharmacological vasodilation enhanced overall oscillatory activity while preserving rhythmic behavior, whereas inhibition of endothelial-derived hyperpolarizing factor reduced oscillation amplitude without eliminating rhythmicity. 

Together, these findings indicate that while vasomotion originates from intrinsic smooth muscle activity, endothelial factors play an important modulatory role by influencing the strength of oscillations. At the network level, this interaction may contribute to the regulation and distribution of microvascular blood flow.

Endothelial and Astrocytic Modulation 

Using acute brain slices and awake in vivo imaging, Haidey et al. [30] investigated the role of astrocytes in very low-frequency arteriolar vasomotion. Increases in vascular tone were associated with calcium elevations in adjacent astrocyte endfeet, suggesting that astrocytes respond to ongoing arteriolar activity rather than initiating rhythmic diameter changes. 

Manipulation of astrocyte signaling pathways further demonstrated a modulatory role. Inhibition or genetic reduction of cyclooxygenase-1 (COX-1) enhanced the power of spontaneous vasomotion without altering baseline vessel diameter, indicating increased oscillatory activity independent of tonic vascular tone. Conversely, reducing astrocyte calcium signaling led to a decrease in vasomotion amplitude and overall oscillatory power, while leaving higher-frequency vascular dynamics largely unaffected. 

Together, these findings suggest that astrocytes do not serve as the primary drivers of vasomotion but instead regulate the strength of oscillatory activity generated by arteriolar smooth muscle. This modulatory role highlights the importance of neurovascular interactions in shaping microvascular dynamics.

Vasomotion in Peripheral Arterial Disease 

Using laser Doppler flowmetry combined with generalized wavelet analysis, Rossi et al. [31] examined rhythmic microvascular flow patterns in healthy individuals and patients with stage II peripheral arterial obstructive disease (PAOD). Resting perfusion was similar between groups, indicating that baseline flow may be preserved despite underlying vascular disease. 

Spectral analysis revealed organized low-frequency oscillations corresponding to vascular smooth muscle, endothelial, and sympathetic activity. Patients with PAOD exhibited increased oscillatory power across several frequency bands, particularly those associated with smooth muscle and endothelial function, suggesting altered microvascular regulation in disease states. 

During post-occlusive reactive hyperemia, healthy individuals demonstrated a coordinated increase in oscillatory activity, whereas patients with PAOD showed a blunted response despite achieving comparable peak perfusion. This finding indicates that while overall blood flow may be restored, the underlying rhythmic organization of microvascular activity remains impaired. 

Consistent with these observations, studies in healthy humans demonstrate that rhythmic microvascular flow patterns are present under resting conditions and follow stable, organized patterns, although the cellular source of these rhythms cannot be determined from human recordings alone [32]. 

Interpretation of these findings should consider methodological differences across studies. In laser Doppler-based studies, oscillations reflect changes in blood flow rather than direct measurements of vessel diameter and may therefore be influenced by factors beyond arteriolar tone. In contrast, imaging studies quantify diameter oscillations directly. Differences in measurement approaches and frequency band definitions limit direct comparison across studies. 

Neural and Hemodynamic Modulation 

There were three included studies that focused on the neural, sympathetic, and hemodynamic factors contributing to the regulation of microvasculature vasomotion [33-35]. Each study investigated different vascular territories and organisms. 

Vetri et al. [33] investigated neurovascular coupling in pial arterioles during sciatic nerve stimulation and observed region-specific changes in oscillatory behavior. While baseline vasomotion patterns were similar across cortical regions, stimulation induced prolonged alterations in oscillatory activity in the hindlimb cortex, suggesting that these oscillatory phenomena occur outside of respiratory control and are due to neurogenic influence (i.e., GABAergic interneurons), ultimately modulating the overall blood flow response in the area. 

Sakurai and Terui [34] demonstrated that sympathetic nerve stimulation influences flow motion without directly altering its intrinsic frequency, indicating that neural input modulates rather than generates oscillatory activity. Environmental factors such as temperature further affected flow motion characteristics, highlighting the sensitivity of vasomotion to systemic conditions. In addition, the presence of flow motion was associated with increased tissue fluid exchange, suggesting a functional role in enhancing microvascular transport. 

Wu et al. [35] demonstrated that physical activity modulates microvascular oscillatory patterns in a workload-dependent manner. Using wavelet analysis of laser Doppler recordings during treadmill walking, the authors identified distinct frequency bands associated with cardiac, respiratory, myogenic, neurogenic, and endothelial activity. Increasing walking intensity led to progressive increases in overall skin blood flow, accompanied by a preferential enhancement of oscillatory power within the neurogenic frequency range. 

Higher walking speeds were associated with a more pronounced contribution of neurogenic oscillations relative to other frequency components, suggesting increased sympathetic involvement at greater metabolic demand. In contrast, endothelial and respiratory-related oscillations showed comparatively smaller changes. These findings indicate that vasomotion is dynamically regulated during exercise, with neural mechanisms playing a dominant role in adjusting microvascular perfusion in response to increasing workload. 

Together, these studies suggest that while vasomotion originates from intrinsic vascular mechanisms, it is strongly modulated by neural input and systemic factors. This interaction enables the microcirculation to adjust oscillatory behavior in response to physiological and environmental demands. 

Coordination and Synchronization of Vasomotion

Five included studies reported findings relevant to the physiology of microvascular vasomotion coordination. 

Zhang et al. [36] investigated mural pial arteriole diameter in vivo and in freshly dissected mouse brains. The authors demonstrated that vasomotor activity is preserved across experimental conditions and measurement approaches, supporting the reliability of diameter-based assessments of vasomotion. Their findings also showed an inverse relationship between smooth muscle Ca²⁺ dynamics and arteriolar diameter changes, reinforcing the role of calcium oscillations as a primary driver of coordinated vasomotor activity. 

In a coronary model, pharmacologic manipulation of nitric oxide and purinergic signaling altered the distribution and organization of flow oscillations, suggesting that endogenous vasodilators contribute to maintaining coordinated and structured vasomotor patterns [37]. Disruption of these pathways resulted in a redistribution of oscillatory activity, consistent with increased irregularity in microvascular flow dynamics. 

Two additional studies, one by Tigno et al. [38] and one by Liu et al. [39], quantified changes in vasomotion complexity and spectral organization using laser Doppler flowmetry, defining oscillations within different frequency ranges as a result of different physiological controls of vasomotion (endothelial, neurogenic, myogenic, respiratory, and cardiac control). Although each study both defined the frequency ranges differently and studied different areas of the microvasculature of different organisms, they both concluded that the nonlinearity [39] or complexity [38] of these oscillatory findings contribute to microvasculature vasomotion events.

In addition to biochemical regulation, temporal factors also influence vasomotor coordination. The fifth study in this category, carried out by Hald et al. [40], investigated the effects of stimulation sequence and timing on vasomotor responses of mesenteric arteries of 48 Sprague-Dawley rats. Exposure to vasoconstrictors induced sustained contraction with superimposed rhythmic oscillations that became more pronounced over time. The subsequent application of vasodilatory stimuli produced relaxation responses that depended on the duration of prior constriction, with longer exposure resulting in greater vasodilatory effects. 

These findings indicate that vasomotion is not only regulated by the type of stimulus but also by the timing and sequence of vascular signals. This temporal dependence suggests that prior vascular activity can influence subsequent oscillatory behavior, contributing to the dynamic and adaptive coordination of vasomotion. 

*Vasomotion in Hypoxia, Ischemia, and Metabolic Stress* 

Five studies examined alterations in microvascular vasomotion under conditions of hypoxia, ischemia, stroke, and metabolic stress using laser Doppler-based, optical, or fluorescence techniques to characterize oscillatory blood flow dynamics. 

Experimental and environmental studies demonstrate that hypoxia enhances vasomotor activity. In both lowlanders exposed to high altitude and native highlanders, slow-wave vasomotion increased with reduced oxygen availability, indicating that hypoxia amplifies oscillatory microvascular activity. In contrast, short-term hypoxia under controlled laboratory conditions did not produce the same baseline effect, suggesting that sustained or systemic hypoxic exposure may be required to induce robust vasomotor responses [41]. 

Clinical studies in patients with acute ischemic stroke show both reduced tissue perfusion and altered vasomotor patterns. Compared with control populations, stroke patients exhibited lower baseline microvascular flow but increased variability and enhanced oscillatory activity across multiple frequency bands, particularly within myogenic and neurogenic ranges [42,43]. In addition, asymmetry in oscillatory patterns between affected and unaffected regions suggests localized disruption of microvascular regulation following ischemic injury. 

In healthy adults, Thorn et al. [44] examined the relationship between vasomotion and oxygen extraction using combined optical reflectance spectroscopy and laser Doppler fluximetry in male participants. Low-frequency oscillations were associated with transient increases in blood flow followed by periods of stable perfusion during which oxygen extraction continued, indicating that vasomotion may facilitate efficient oxygen delivery at the tissue level.

Further evidence suggests that baseline vasomotor activity reflects the capacity for microvascular adaptation to hypoxia. Individuals with stronger baseline oscillatory activity demonstrated more robust responses to transient hypoxic stress, whereas reduced baseline vasomotion was associated with impaired adaptive responses [45]. 

Together, these findings indicate that vasomotion is dynamically modulated under hypoxic and ischemic conditions and may play an important role in regulating microvascular perfusion and oxygen delivery.

*Mechanical and Shear-Dependent Mechanisms* ​​​​

Beyond cellular and neural inputs, the origin of microvascular vasomotion is fundamentally influenced by mechanical forces, hemodynamic shear stress, and the microvascular network's structural architecture. 

Local heating-induced shear responses were examined in the thermoregulatory microcirculation of the pallid bat wing using intravital microscopy [46]. Local heating produced a biphasic increase in arteriolar blood flow, with smaller arterioles responding more rapidly than larger vessels. These changes were associated with transient increases in endothelial shear stress, followed by stabilization as the vessel diameter adapted. Pharmacologic inhibition of sensory neural signaling and nitric oxide pathways attenuated these responses, indicating that both shear stress and endothelial signaling contribute to coordinated vasomotor activity.

Network-level organization further influences vasomotion. In a subsequent study with the same bat wing preparation, upstream metarterioles were identified as sites exhibiting phase-leading oscillatory activity for downstream vessels [47]. Diameter measurements demonstrated that oscillations in upstream microvessels occurred before those observed in smaller downstream branches, indicating coordinated propagation within the microvascular network. This pattern was reproducible across repeated heating trials, suggesting hierarchical organization rather than random local fluctuations. These findings support the presence of spatially organized microvascular coordination within resistance-sized arterioles, with upstream segments contributing to the timing and synchronization of downstream vasomotor responses. 

Mechanical and environmental factors may also alter vasomotion characteristics. In a rat model of caudal artery ligation, changes in oscillatory activity were observed without corresponding alterations in mean perfusion, indicating that vasomotion can be modulated independently of baseline blood flow [48]. External physical influences, such as magnetic field exposure, were associated with changes in oscillation amplitude, further supporting the sensitivity of vasomotion to mechanical and environmental conditions. 

Together, these findings highlight the importance of mechanical forces and vascular architecture in shaping vasomotion, complementing cellular and biochemical mechanisms to regulate microvascular function.

*Computational and Theoretical Models* 

Various conceptual and computational models have been developed to explain how vasomotor oscillations emerge in the microvasculature and how they are coordinated across vessel networks. 

Early models focused on the intrinsic myogenic response of vascular smooth muscle and endothelial cells. One model examined the relationship between transmural pressure and vessel wall stress, demonstrating that increases in pressure increase intracellular Ca²⁺ concentration, leading to contraction and the emergence of oscillatory behavior within a defined range of pressure and agonist levels that supported oscillatory behavior [22]. Oscillation frequency varied according to the timing of the pressure change within the cycle and the underlying calcium signaling dynamics. The model predicted that oscillatory behavior was more pronounced in smaller resistance vessels and diminished in larger arteries, reflecting differences in wall thickness and mechanical responsiveness. 

Similarly, ion-based models examined the contribution of potassium and calcium fluxes to spontaneous membrane potential oscillations in vascular smooth muscle cells [12]. These models demonstrated that interactions between voltage-dependent calcium channels and potassium conductance can produce periodic membrane depolarizations, resulting in rhythmic contraction. Oscillation frequency and stability were sensitive to changes in ion channel conductance parameters, indicating that vasomotion can arise from intrinsic electrophysiological properties of smooth muscle cells. 

Building on models of intracellular calcium dynamics, other models have focused on how signaling pathways coordinate oscillatory behavior between SMCs [49]. In a single-cell oscillator framework, cyclic guanosine monophosphate (cGMP) signaling was shown to influence synchronization behavior. Elevated cGMP levels enhanced intracellular Ca²⁺ cycling and increased oscillation amplitude, promoting vasomotor activity. Reduced cGMP levels were associated with desynchronized oscillations and altered frequency, highlighting the role of membrane potential and second-messenger signaling in regulating rhythmic behavior. 

In contrast to models centered on intrinsic cellular oscillators, other approaches emphasized vessel-level mechanical and metabolic feedback as drivers of vasomotion [50]. Single-vessel models incorporating wall tension, shear stress, and oxygen-dependent metabolic signals demonstrated that spontaneous oscillations emerge from dynamic interactions between vascular tone and mechanical feedback. It was reported that these oscillations arise from interactions between the vessel wall and its response to wall tension rather than from pre-specified intrinsic cell oscillators. 

Computational modeling studies have also examined how vasomotion influences tissue oxygenation at the network level. In a two-dimensional model of skeletal muscle consisting of interleaved capillaries and muscle fibers, Pradhan and Chakravarthy [51] simulated interacting vasomotor units. Their model demonstrated that oxygen delivery was not optimized under fully synchronized vasomotion. Instead, oxygenation was greatest when both the neuronal network and the vasomotor network exhibited desynchronized activity. 

Discussion 

This scoping review synthesizes experimental, clinical, and computational evidence examining the cellular and biophysical origins of microvascular vasomotion. Across various species and methodological approaches, the findings support the concept that vasomotion is primarily driven by intrinsic oscillatory activity within arteriolar vascular smooth muscle cells (SMCs), with additional modulation from endothelial, neural, metabolic, and mechanical influences. While individual studies differ in design and analytic methods, a mechanistic pattern emerges. The inclusion of vessels up to 100 µm reflects the functional definition of the microcirculation, particularly the role of resistance arterioles as the primary site of vasomotion generation. 

*Smooth Muscle Calcium Oscillations as a Primary Driver* 

The most consistent evidence across included studies suggests that rhythmic changes in intracellular Ca²⁺ within arteriolar SMC serve as the primary driver of vasomotion. 

Direct in vivo imaging provides strong support for intrinsic smooth muscle oscillators. Li et al. [25] demonstrated that transient ischemia significantly suppressed SMC Ca²⁺ oscillation frequency and amplitude while leaving mean vessel diameter unchanged, indicating that Ca²⁺ dynamics regulate rhythmic fluctuations rather than baseline tone. Similarly, Borysova et al. [26] showed that higher-frequency and synchronized Ca²⁺ oscillations were associated with stronger and more coordinated microvascular constrictions, whereas lower-frequency activity produced weaker contractions. Together, these findings suggest that both the frequency and synchronization of Ca²⁺ signaling determine the strength of vasomotion. 

Earlier intravital observations by Colantuoni et al. [3] demonstrated segmental localization of oscillations and an inverse relationship between vessel diameter and oscillation frequency, supporting the concept of locally generated oscillators within resistance-sized arterioles. Electrophysiologic findings further reinforce this interpretation. Yang et al. [28] identified vessel-specific bimodal resting membrane potential distributions that were sensitive to Kir channel blockade, suggesting that intrinsic membrane conductance properties shape rhythmic electrical behavior. Importantly, differences were functional rather than expression-based, indicating that channel dynamics, rather than channel abundance, determine oscillatory stability. 

Molecular manipulation studies clarify the importance of intercellular coordination. Broegger et al. [27] showed that bestrophin-3 downregulation impaired vasomotion amplitude and synchronization without altering tonic contraction, indicating that intrinsic oscillations persist but require coordinated coupling for full expression. This finding suggests that synchronized oscillatory activity, not just an elevation in calcium, is necessary for full vasomotor expression. 

Collectively, imaging, electrophysiological, and molecular data support a model in which intrinsic SMC Ca²⁺ oscillations generate the foundational vasomotor rhythm, while membrane properties and intercellular coupling determine synchronization strength. 

Endothelial and Astrocytic Modulation 

Although smooth muscle calcium oscillations appear to drive vasomotion, endothelial and astrocytic signaling shape their expression. 

Endothelial signaling does not appear to initiate oscillations, but it significantly influences their amplitude and spectral distribution. Pharmacologic disruption of endothelial-derived hyperpolarizing pathways reduces oscillation strength while preserving rhythmicity [29], suggesting that endothelial input fine-tunes vasomotor expression rather than serving as a pacemaker. This distinction is important, as it separates rhythm generation from amplitude modulation. 

Similarly, astrocytic signaling in the cerebral microcirculation appears to regulate oscillatory power without independently generating rhythmic diameter changes [30]. Astrocytes respond dynamically to vascular tone and influence low-frequency oscillatory strength, but intrinsic smooth muscle activity persists even when astrocytic signaling is reduced. These findings support a hierarchical model in which smooth muscle provides the oscillatory core, while perivascular cellular elements adjust the magnitude and regional coordination of that activity. 

Together, endothelial and astrocytic influences appear to function as modulators of an existing oscillator rather than primary drivers of vasomotion. 

Neural and Sympathetic Contributions 

Neural and sympathetic activity also influence vasomotion, although they do not appear to be its primary sources. Experimental stimulation studies and exercise models consistently demonstrate that neurogenic activation enhances oscillatory prominence without fundamentally altering intrinsic oscillation frequency [33-35]. 

Rather than generating new rhythmic patterns, sympathetic activity appears to amplify or redistribute power within established frequency bands. This suggests that neural input adjusts oscillatory strength and spatial distribution in response to metabolic or functional demand. Importantly, vasomotion persists even in the absence of acute neural stimulation, reinforcing the concept that the oscillator resides within the vessel wall itself. 

Regional variability also emerges across vascular beds, indicating that neurovascular modulation is context-dependent. Cerebral, cutaneous, and peripheral microcirculations do not respond identically to stimulation, further supporting the concept of locally generated oscillators subject to bed-specific modulation. 

From a clinical perspective, this distinction is meaningful. Vasomotion can persist even when acute neural input is reduced while remaining responsive to shifts in sympathetic tone. Together, these findings indicate that neural signals adjust the strength and pattern of vasomotion but are not its primary source. 

Vasomotion Coordination, Synchronization, and Network Dynamics 

In addition to how vasomotion is generated within individual SMCs, several studies highlight the importance of its coordination across the vascular network. Vasomotion does not occur as isolated contractions in single cells but rather as synchronized activity across groups of neighboring arterioles. 

Studies show that vasomotion follows organized frequency patterns rather than random fluctuations. When signaling pathways such as nitric oxide or purinergic signaling are altered, the strength and distribution of oscillations change, but rhythmic activity often persists. This suggests that these pathways influence how oscillations are organized and expressed across vessels rather than determining whether they occur at all [36-40]. 

Experimental findings also indicate that the timing of stimulation affects the strength and pattern of vasomotion. In some models, vessels exposed to the same stimulus at different time intervals demonstrate different oscillatory responses [40]. This supports the idea that vasomotion depends not only on individual smooth muscle activity but also on how cells interact with each other within the network. 

Importantly, when synchronization between cells is disrupted, oscillations may weaken or become more irregular, even though rhythmic activity is not completely lost [27]. This suggests that coordination mechanisms determine the strength and consistency of vasomotion, while the underlying rhythm is governed by smooth muscle activity. 

Vasomotion in Hypoxia, Ischemia, and Metabolic Stress 

Across conditions of arterial occlusion, intermittent hypoxia, and acute ischemic stroke, vasomotion was often observed to have increased in strength. Several studies report amplification of low-frequency oscillations during high-altitude exposure, arterial occlusion, and acute ischemic stroke [41-43,45]. 

This amplification often occurs without proportional changes in mean perfusion, suggesting that oscillatory enhancement may represent a regulatory adaptation rather than simply a reflection of altered flow. One interpretation, supported by experimental and modeling studies, is that increased rhythmic activity promotes more dynamic redistribution of microvascular perfusion, potentially supporting oxygen extraction during metabolic stress [41,44,51]. However, it remains unclear whether enhanced oscillatory behavior represents compensation or early dysfunction. 

Clinical observations in stroke and peripheral arterial disease demonstrate altered oscillatory organization and variability [31,42,43], further suggesting that vasomotion characteristics reflect underlying microvascular health. Whether these changes are protective, maladaptive, or neutral markers remains unresolved. 

These findings may also have implications for microvascular perfusion. When normal vasomotor patterns are disrupted, blood flow may become unevenly distributed, which can limit effective oxygen delivery even when overall perfusion appears adequate. This may be relevant in conditions such as the no-reflow phenomenon, where microvascular function remains impaired despite restoration of upstream blood flow [52]. In this setting, altered vasomotion may contribute to reduced capillary perfusion and persistent tissue hypoxia. 

From a clinical perspective, the ability to assess vasomotor patterns using non-invasive techniques such as laser Doppler flowmetry or optical imaging suggests potential applications in evaluating microvascular function. In addition, a better understanding of the mechanisms underlying vasomotion may help inform future strategies aimed at improving microvascular regulation in disease states.

Mechanical, Shear, and Structural Drivers 

Mechanical forces within the vessel wall contribute to the expression and propagation of vasomotion. Shear stress, transmural pressure, and wall tension interact with intrinsic smooth muscle activity to shape oscillatory behavior [22,46,47]. 

Experimental studies demonstrate that upstream resistance vessels may begin oscillatory cycles earlier than downstream branches, indicating coordinated propagation within the microvascular network. These findings indicate that mechanical and structural factors influence the spatial coordination of oscillations, even if they do not initiate the underlying rhythm. 

Computational models reinforce this perspective by showing that oscillations can emerge from dynamic interactions between pressure-dependent myogenic responses and wall stress feedback [50]. Rather than acting independently, mechanical forces appear to interact with intrinsic smooth muscle oscillators to stabilize or amplify rhythmic behavior. 

Conceptual and Computational Models of Vasomotion 

Theoretical and computational models provide additional support for a hybrid framework of vasomotion. Ion-based models demonstrate that interactions between voltage-dependent calcium channels and potassium conductance can generate periodic depolarizations consistent with intrinsic smooth muscle oscillators [12,49]. Other models emphasize vessel-level feedback mechanisms in which wall tension and shear stress contribute to oscillatory emergence [22,50]. 

Network simulations suggest that fully synchronized oscillations may not optimize oxygen delivery, whereas partial desynchronization may enhance tissue oxygenation [51]. Although these models examine individual mechanisms in isolation, taken together with experimental findings, they support the view that vasomotion originates within smooth muscle cells and is shaped by mechanical forces and metabolic conditions in the surrounding network. 

*Clinical Implications* 

Although many of the included studies are experimental, vasomotion is consistently measurable in humans using non-invasive techniques such as laser Doppler flowmetry and related optical methods. Rhythmic fluctuations in microvascular blood flow can be detected under resting conditions and during physiologic stress, allowing assessment of microvascular dynamics in clinical settings. 

Alterations in vasomotor patterns have been observed in patients with acute ischemic stroke, peripheral arterial disease, and during hypoxic exposure. In these contexts, changes in the strength, regularity, or organization of oscillations have been reported even when overall perfusion appears relatively preserved. This suggests that vasomotion analysis may provide information about microvascular regulation that is not captured by static measures of blood flow alone. 

Given that vasomotion reflects interactions between arteriolar smooth muscle activity, endothelial function, neural input, and mechanical forces, abnormalities in oscillatory behavior may serve as an early indicator of microvascular dysfunction. Changes in vasomotion characteristics could potentially help identify impaired vascular responsiveness before overt structural disease develops. 

However, the clinical role of vasomotion assessment remains to be fully defined. It is not yet clear whether specific oscillatory patterns reliably predict disease progression, therapeutic response, or tissue oxygenation efficiency. Standardization of measurement techniques and frequency definitions will be necessary before vasomotion can be incorporated into routine clinical evaluation. 

Limitations 

This review has several limitations. First, the included studies were highly heterogeneous in terms of the species studied, the vascular territories examined, and the measurement techniques used. Differences in how frequency ranges were defined and analyzed make direct comparison across studies challenging and limit the ability to draw quantitative conclusions. Additionally, differences between direct measurements of vessel diameter and flow-based assessments (laser Doppler flowmetry) complicate interpretation, as flow oscillations may not directly reflect underlying arteriolar vasomotion.

In human studies, vasomotion is typically assessed using indirect measures of blood flow fluctuations rather than direct imaging of smooth muscle calcium activity. As a result, the precise cellular source of oscillations cannot always be confirmed in clinical settings. While frequency-based interpretations are widely used, they rely on assumptions that may not fully capture underlying mechanisms. 

Additionally, many experimental models isolate specific pathways, such as calcium signaling or mechanical feedback, in order to clarify individual mechanisms. While this approach improves mechanistic understanding, it may oversimplify the complex and integrated regulation that occurs in intact vascular networks. 

These limitations highlight the need for standardized measurement approaches and integrative experimental designs to better define the origins and clinical significance of vasomotion. 

*Future Directions* 

Future research should focus on integrating mechanistic and clinical approaches to better define the role of vasomotion in health and disease. Simultaneous measurement of intracellular calcium activity and microvascular blood flow in intact vascular beds would help clarify how cellular oscillations translate into network-level hemodynamic changes. Greater standardization in the definition and reporting of frequency ranges across studies would also improve comparability and facilitate translation to clinical settings. In addition, further investigation is needed to better understand how mechanical feedback within the vessel wall interacts with intrinsic smooth muscle oscillations. Finally, longitudinal clinical studies are necessary to determine whether changes in vasomotion can serve as reliable biomarkers of vascular dysfunction, disease progression, or treatment response. Improved integration of experimental and clinical data may help clarify whether alterations in vasomotion represent adaptive regulation or early evidence of microvascular impairment. 

## Conclusions

This review mapped the current evidence regarding the origins of microvascular vasomotion and its physiological effects. Across 30 studies, a consistent mechanistic pattern emerged: vasomotion appears to arise primarily from intrinsic oscillatory activity within arteriolar smooth muscle cells, particularly rhythmic intracellular Ca²⁺ dynamics, with coordination shaped by membrane electrophysiology, ion channel behavior, and intercellular coupling. Endothelial, astrocytic, neural, metabolic, and mechanical influences were repeatedly shown to modulate the amplitude, synchronization, and spatial organization of oscillations, but not to serve as the fundamental pacemaker in most models. However, substantial heterogeneity remains in experimental design, vascular territories studied, and frequency-band definitions, limiting direct cross-study comparison. This review provides a structured synthesis of mechanistic pathways and highlights key gaps, including the need for integrative in vivo models that simultaneously assess cellular oscillations and network-level flow dynamics. Future work should prioritize standardized experimental design, translational validation in human microvascular beds, and longitudinal clinical studies to determine whether vasomotion can serve as a reliable biomarker of microvascular health. 
